# The Dawn of Lead‐Free Perovskite Solar Cell: Highly Stable Double Perovskite Cs_2_AgBiBr_6_ Film

**DOI:** 10.1002/advs.201700759

**Published:** 2017-12-18

**Authors:** Cuncun Wu, Qiaohui Zhang, Yang Liu, Wei Luo, Xuan Guo, Ziru Huang, Hungkit Ting, Weihai Sun, Xinrui Zhong, Shiyuan Wei, Shufeng Wang, Zhijian Chen, Lixin Xiao

**Affiliations:** ^1^ Laboratory for Mesoscopic Physics and Department of Physics Peking University Beijing 100871 P. R. China; ^2^ Co‐Innovation Center for Micro/Nano Optoelectronic Materials and Devices Chongqing University of Arts and Sciences Yongchuan Chongqing 402160 P. R. China; ^3^ New Display Device and System Integration Collaborative Innovation Center of the West Coast of the Taiwan Strait Fuzhou 350002 P. R. China

**Keywords:** Cs_2_AgBiBr_6_, double perovskites, lead‐free, perovskite solar cell, planar heterojunction

## Abstract

Recently, lead‐free double perovskites have emerged as a promising environmentally friendly photovoltaic material for their intrinsic thermodynamic stability, appropriate bandgaps, small carrier effective masses, and low exciton binding energies. However, currently no solar cell based on these double perovskites has been reported, due to the challenge in film processing. Herein, a first lead‐free double perovskite planar heterojunction solar cell with a high quality Cs_2_AgBiBr_6_ film, fabricated by low‐pressure assisted solution processing under ambient conditions, is reported. The device presents a best power conversion efficiency of 1.44%. The preliminary efficiency and the high stability under ambient condition without encapsulation, together with the high film quality with simple processing, demonstrate promise for lead‐free perovskite solar cells.

Organic–inorganic hybrid perovskites for photovoltaic cells have drawn enormous attention in recent years due to their excellent optical and electronic properties.^1^, ^2^, ^3^, ^4^, ^5^, ^6^, ^7^, ^8^, ^9^, ^10^, ^11^ Nowadays, the highest power conversion efficiency (PCE) of perovskite based solar cells has already reached over 22%,^12^ outperforming several other types of third‐generation solar cells. It becomes the most promising candidate for next‐generation of photovoltaic devices after the conventional silicon solar cells. However, for the typical APbX_3_ (A = CH_3_NH_3_ (MA), (NH_2_)_2_CH (FA), Cs, X = Cl, Br, I) perovskite‐based solar cells, the stability and toxicity are the key concerns for their commercialization.^13^, ^14^

For lead‐free perovskite solar cells (PSCs), tin is at the focus owing to a similar diameter and valence to lead, which prompts people to substitute lead with tin to form ASnX_3_ perovskite films.^15^, ^16^, ^17^, ^18^, ^19^, ^20^, ^21^ However, the highest PCE of tin‐based device achieved was only 6.4%,^17^ which is lower than lead‐based perovskite solar cell. More importantly, Sn^2+^ ion in the material is unstable and easily to be oxidized to Sn^4+^, leading to a destruction of photovoltaic performance. It is still difficult to develop new lead‐free perovskites with good intrinsic stability for photovoltaic applications.

An alternative way to expand the perovskite family for photovoltaic application is to replace two divalent Pb^2+^ ions with one monovalent M^+^ and one trivalent M^3+^ ions, forming A_2_M^+^M^3+^X_6_ double perovskite structure named elpasolite.^22^ It is a rich family (more than 350 different elpasolites have been synthesized^23^) of novel lead‐free perovskite. Through first‐principles calculations, eleven optimal materials were found with suitable bandgap as photovoltaic absorbers. However, only three of them are synthesized till now, including Cs_2_AgBiBr_6_,^24^ Cs_2_AgBiCl_6_,^25^ and (CH_3_NH_3_)_2_AgBiBr_6_.^26^ Bandgap engineering of Cs_2_AgBiBr_6_ had been operated by defect inducing^27^ and trivalent metal alloying.^28^ By adding 0.075% Tl (I), a direct bandgap of 1.57 eV could be obtained in Cs_2_AgBiBr_6_ 27 and 1.86 eV for Cs_2_Ag(Bi_0.625_Sb_0.375_)Br_6_,^28^ which show great potential in solar cell applications. However, though this double perovskite is quite stable as lead‐free perovskite, to the best of our knowledge, no solar cell has yet been reported, because of the difficulties in film formation. A lead‐free perovksite base solar cell with high performance, high stability, and solvent processibility cannot be reached at moment.

In this work, we successfully prepared high quality, highly stable double perovskite Cs_2_AgBiBr_6_ film by a low‐pressure assisted (LPA) method. Planar heterojunction solar cells based on this film were fabricated. The annealing effects on the crystallinity and photovoltaic properties were studied systematically. The optimized PCE is up to 1.44%. This is significantly higher than the traditional thermally annealed (TA) double perovskite solar cells. We attribute the improvement on PCE to the significantly improved Cs_2_AgBiBr_6_ film quality by the LPA method. Furthermore, it is the highest PCE for lead‐free (except Sn) perovskite solar cell. This film based device is much more stable than Sn‐based PSCs. For HTM‐free device, the PCE showed no obvious degradation after four months under ambient conditions. Our results indicate an important improvement toward the realization of lead‐free perovskite solar cell.

Orange Cs_2_AgBiBr_6_ powder was synthesized according to the previously reported method.^24^ It was dissolved in dimethylsulfoxide (DMSO) at a concentration of 0.5 mol L^−1^ to form a transparent light yellow solution (**Figure** **1**a). Uniform Cs_2_AgBiBr_6_ film was fabricated by an optimized LPA method (similar methods have been reported for organic–inorganic hybrid perovskite film fabrication^29^, ^30^ under ambient conditions. The film fabrication process diagram is illustrated in Figure 1b. In detail, the solution was first spin‐coated on a glass/ITO substrate. The spin‐coated film was quickly moved to a low‐pressure chamber pumped to 20 Pa, in which the transparent film would gradually turn to light yellow. Then it was annealed at 200 °C to evaporate the residual solvent, achieving a uniform film with good crystallinity. On the contrary, the traditional thermal annealing method (TA) generated a low coverage Cs_2_AgBiBr_6_ film and the corresponding devices usually showed a poor PCE (<0.1%, Figure S1, Supporting Information). According to the scanning electron microscope (SEM) images (Figure 1c,d) and the film photographs (the inset of Figure 1c,d), it was obvious that the LPA film showed a dense and smooth morphology. This is clearly different to the TA film, which has many insular particles. This result indicates that the LPA process is crucial to the formation of Cs_2_AgBiBr_6_ film.

**Figure 1:**
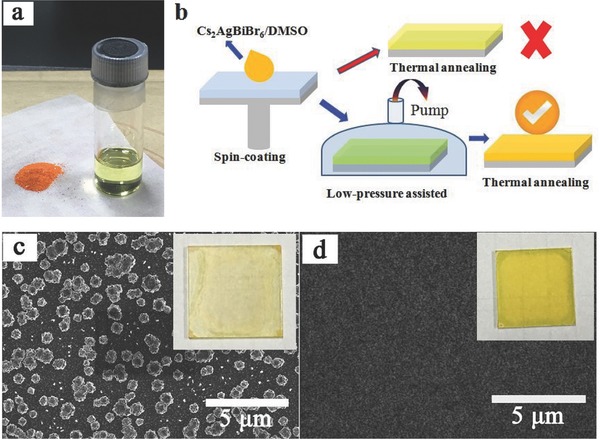
Fabrication and SEM images of Cs_2_AgBiBr_6_ film. a) Image of Cs_2_AgBiBr_6_ powder (left) and solution in DMSO (right). b) The film fabrication process diagram. c,d) SEM images of film obtained by c) TA and d) LPA process, inset: film photograph, size, 25 mm × 25 mm.

To characterize, first, the Cs_2_AgBiBr_6_ film was deposited on a quartz glass with LPA method. Its crystalline structure was identified by X‐ray diffraction (XRD). The diffraction peaks are indexed as (111), (200), (220), (311), (222), (400), (420), (422), (440), (620), (444), (642), (800), and (840) planes of cubic crystalline structure of Cs_2_AgBiBr_6_ (**Figure** **2**a). The cell unit parameter is refined to *a* = 11.2640(8) Å with the space group *Fm‐3m*, which agrees to the Cs_2_AgBiBr_6_ powder.^24^ A schematic picture of this double perovskite structure is shown in Figure 2b. The bandgap of Cs_2_AgBiBr_6_ powder extracted from ultraviolet visible (UV–vis) absorption spectrum (Figure S2, Supporting Information) is 2.05 eV, which agrees with the literature result.^24^ However, for Cs_2_AgBiBr_6_ film, a strong absorption peak was observed at 438 nm, with a weaker tailing absorption edge to 610 nm (Figure 2c). The peak at 438 nm might be attributed to the exciton absorption, from which an exciton binding energy of 220 meV can be estimated. A similar absorption spectra were also observed in Cs_3_Bi_2_I_9_.^31^ The exciton binding energy value of Cs_2_AgBiBr_6_ is much larger than that of CH_3_NH_3_PbI_3_ (25 meV), which might be one of the reason for the lower PCE of Cs_2_AgBiBr_6_ device. Steady‐state photoluminescence (PL) spectrum shows that Cs_2_AgBiBr_6_ film has relatively broad PL emission, with the peak centered at 615 nm (2.01 eV). In addition, the valence band maximum of this film estimated from ultraviolet photoelectron spectrum (Figure S3, Supporting Information) is 6.22 eV, which is close to the value in literature.^24^

**Figure 2:**
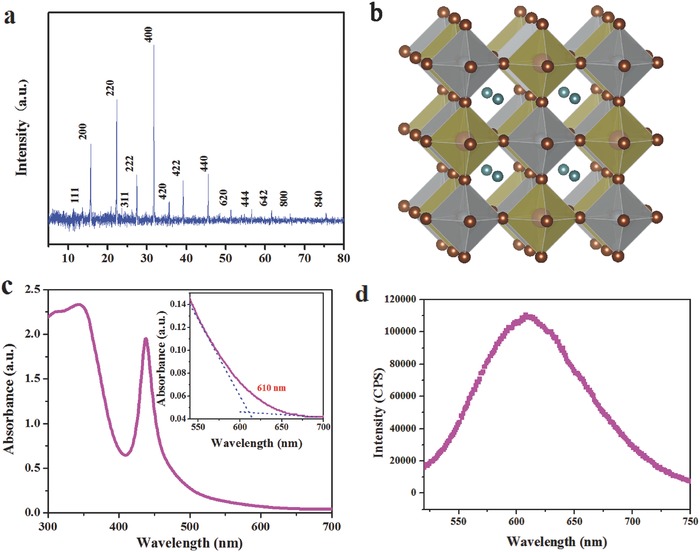
XRD pattern, crystal structure, optical absorption, and steady‐state photoluminescence. a) X‐ray diffraction pattern of Cs_2_AgBiBr_6_ film. b) Refined crystal structure diagram of Cs_2_AgBiBr_6_. The Br^−^ ions are shown as dark red spheres, the Cs^+^ ions are shown as turquoise, while Ag and Bi centered octahedral are shown as gray and brown polyhedral, respectively. c) Absorption spectrum of Cs_2_AgBiBr_6_ film. d) Steady‐state photoluminescence spectrum of Cs_2_AgBiBr_6_ film.

To investigate the influence of annealing temperature, Cs_2_AgBiBr_6_ films were deposited onto FTO/SnO_2_ substrates by an LPA method with different annealing temperature of 150, 250, and 300 °C, respectively. **Figure** **3**a–c presents the surface scanning electron microscopy (SEM) images of them. It can be observed that all the substrates were well covered by dense and uniform Cs_2_AgBiBr_6_ films, without any visible pinholes. The grain size of the 150 °C annealed film is within a few tens of nanometers. However, for the films annealed at 250 °C or 300 °C, more uniform morphology and larger grain size were observed. XRD of Cs_2_AgBiBr_6_ film deposited on FTO/SnO_2_ was measured to identify the crystallinity of Cs_2_AgBiBr_6_, as shown in Figure 3d. Beside the diffraction peaks from FTO/SnO_2_ substrate, all the other diffraction peaks well agree the result of Cs_2_AgBiBr_6_ film on top of quartz glass. No additional diffraction peaks were found, indicating the high crystalline phase purity. It is worth noting that the diffraction peaks at preferred orientation of 15.71°(200) and 31.76°(400) become stronger with annealing temperature increasing, suggesting that both the grain size and crystallinity of the Cs_2_AgBiBr_6_ film were enhanced with higher annealing temperature. In addition, no noticeable difference can be observed from their UV–vis absorption spectra with different annealing temperatures (Figure S4, Supporting Information). These results indicate that the thermal stability of double perovskite Cs_2_AgBiBr_6_ is much higher than that of hybrid APbX_3_ perovskite (decomposite at ≈150 °C).

**Figure 3:**
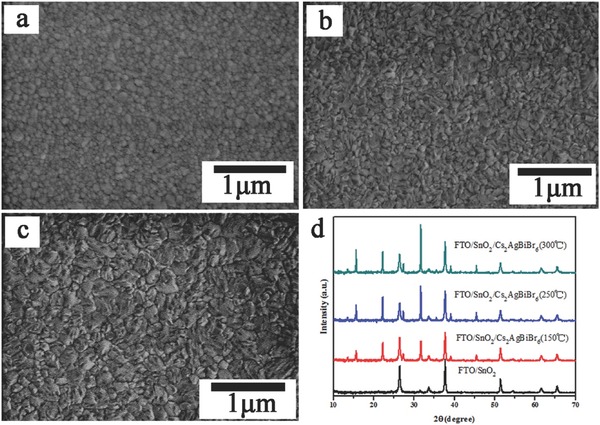
SEM images and XRD patterns of FTO/SnO_2_/Cs_2_AgBiBr_6_ film annealed under different temperatures. SEM images of film from a) 150 °C, b) 250 °C, c) 300 °C. d) XRD patterns of film from 150 °C (red), 250 °C (blue), and 300 °C (green).

To investigate the photovoltaic performance of Cs_2_AgBiBr_6_ double perovskite, a planar n‐i‐p architecture solar cell has been fabricated with SnO_2_ and poly(3‐hexylthiophene‐2,5‐diyl) (P3HT) as the electron and hole transport material (HTM), respectively. The device structure and the corresponding energy alignment diagram are given in **Figure** **4**a,b respectively. Figure 4c shows the cross‐sectional SEM image (the film annealed at 250 °C) of this solar cell. We could estimate that the thickness of Cs_2_AgBiBr_6_ film is 150 nm, which is limited by the solubility of Cs_2_AgBiBr_6_ in DMSO. The photocurrent–voltage performance of the cells annealed under different temperatures is shown in Figure 4d. The corresponding device performances improved with the annealing temperature increasing from 150 to 250 °C. In detail, the open‐circuit voltage (*V*_oc_) is improved from 1.03 to 1.07 V, while the short‐circuit current density (*J*_sc_) is improved from 0.78 to 1.78 mA cm^−2^, with fill factors (FF) from 0.67 to 0.72, and the PCE from 0.57 to 1.37%, respectively. However, the PCE would slightly decrease when the annealing temperature was further increased to 300 °C. Detailed solar cell parameters extracted from the *J*–*V* characteristics are listed in **Table** **1**. We attribute the better performance of high temperature annealing devices to the improved Cs_2_AgBiBr_6_ film quality. It can be inferred that the defect density of Cs_2_AgBiBr_6_ film annealed with high temperature is relatively lower than that of film with low temperature annealing treatment. More detailed discussions are given in the below.

**Figure 4:**
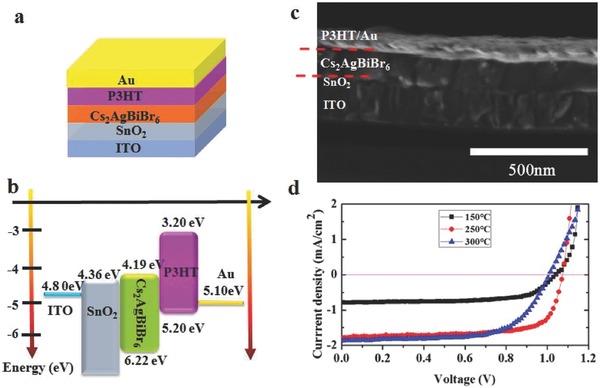
ITO/SnO_2_/Cs_2_AgBiBr_6_/P3HT/Au solar cells. a) Device configuration diagram. b) Schematic of energy alignment diagram. c) Cross‐sectional SEM image of device. d) *J–V* curves under different annealing temperature.

**Table 1:** Device performances of Cs_2_AgBiBr_6_ films annealed under different temperatures

Sample	*J*_sc_ [mA cm^−2^]	*V*_oc_ [V]	FF	PCE (maximum) [%]
Cs_2_AgBiBr_6_ (150 °C)	0.76 ± 0.05	1.02 ± 0.01	0.63 ± 0.04	0.49 ± 0.05 (0.54)
Cs_2_AgBiBr_6_ (250 °C)	1.78 ± 0.03	1.07 ± 0.01	0.69 ± 0.03	1.32 ± 0.05 (1.37)
Cs_2_AgBiBr_6_ (300 °C)	1.79 ± 0.08	0.99 ± 0.02	0.65 ± 0.04	1.16 ± 0.06 (1.22)

The maximum PCE of 1.44% (**Figure** **5**a black line) was achieved in the Cs_2_AgBiBr_6_ film annealed at 250 °C, with *V*_oc_ of 1.04 V, *J*_sc_ of 1.78 mA cm^−2^, and a high FF of 0.78 measured in forward scan. Meanwhile, an HTM‐free device was fabricated with the optimized film, and a PCE of 0.86% (Figure 5a red line) with *V*_oc_ of 0.95 V, *J*_sc_ of 1.50 mA cm^−2^, and FF of 0.60 were obtained. A hysteresis phenomenon was still observed in these two different architecture devices (Figure S5 and Table S1, Supporting Information). To be noted here, the forward scanning PCE for the device with P3HT is larger than that of reverse. This might be attributed to the unbalanced charge transport in the device.

**Figure 5:**
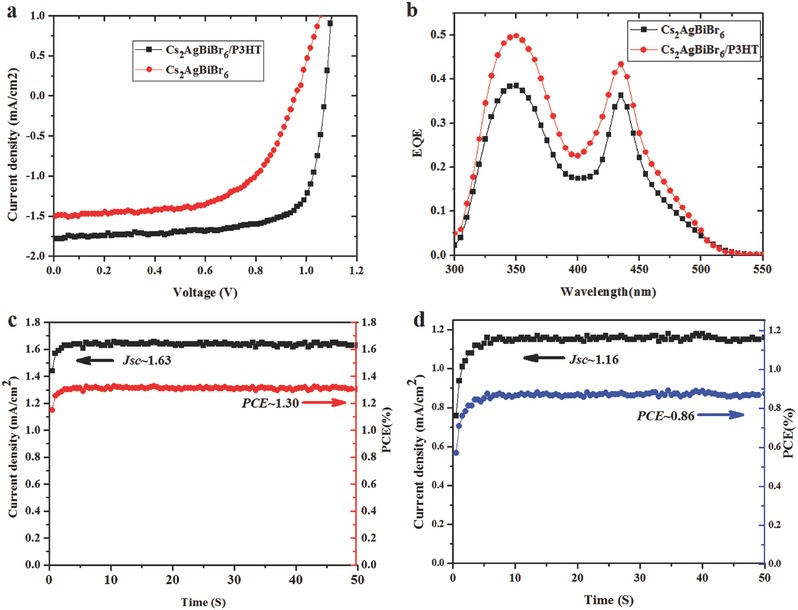
Current–voltage (*J*–*V*) characteristics and steady‐state photocurrent of devices at AM 1.5 illumination, EQE spectrum of Cs_2_AgBiBr_6_ film (with or without P3HT). a) *J*–*V* curves of devices. b) EQE spectrum. Steady‐state output performance of device c) with P3HT and d) without P3HT.

The stabilization of PCEs was estimated by measuring the steady‐state photocurrent at the maximum power point under simulated solar illumination. The solar cell with HTM showed a stabilized PCE of 1.30% at a bias of 0.80 V (Figure 5c), while the HTM‐free device showed 0.86% at a bias of 0.75 V (Figure 5d). These results closely matched the values extracted from the *J*–*V* curve with a forward scan. The external quantum efficiency (EQE) spectra (Figure 5b) were measured to further demonstrate the accuracy of the output *J*_sc_ and afford an integrated photocurrent of 1.70 and 1.31 mA cm^−2^ for the device with or without HTM, respectively, which approximate to the measured *J*_sc_.

Defect often plays a critical role to solar cell performance. To qualitatively assess the defect density of Cs_2_AgBiBr_6_ films, we fabricated ITO/Cs_2_AgBiBr_6_/Au devices, and measured the space charge limited current as shown in Figure S6a–c (Supporting Information). The curve at low bias voltage indicates an Ohmic response of the device. A sharp increase appears when the bias voltage exceeds the trap‐filled limit voltage (*V*_TFL_), demonstrating that the trap states are completely filled. The defect density in the film can be estimated by

(1) $$n_{\mathrm{defects}}=2\varepsilon_{0}\varepsilon V_{\mathrm{TFL}}/qL^{2}$$

where ε_0_ and ε are the vacuum permittivity and the relative dielectric constant of Cs_2_AgBiBr_6_, respectively. *L* is the thickness of the Cs_2_AgBiBr_6_ film, and *q* is the elementary charge. The *V*_TFL_ decreased with annealing temperature increasing, indicating a corresponding decrease in defect density, which is the origin of the improved PCE of Cs_2_AgBiBr_6_ film from a relatively higher temperature annealing treatment.

The carrier recombination process of the working layer was analyzed by time‐resolved photoluminescence (TRPL) measurement (Figure S7, Supporting Information). The data can be fitted with triple exponential,^24^ the decay component of τ_1_ ≈ 1 ns was assigned to bimolecular recombination, while the second one of τ_2_ ≈ 30 ns could be attributed to recombination of trap and/or surface‐state emission. The longest PL decay could be attributed to exciton lifetime. As the fitted data shown in Table S2 (Supporting Information), the τ_2_ ratio of film annealing at 250 °C is the lowest, while the one annealing at 150 °C is the highest, which corresponds to the *V*_oc_ value of the cells with different annealing temperatures. According to the TRPL measurement, the long lifetimes of optimized Cs_2_AgBiBr_6_ film (annealing at 250 °C) provide the possibility to efficiently extract the carriers from working layer.

We also investigated the stability of the double perovskite Cs_2_AgBiBr_6_ solar cells under ambient conditions (temperature: 20–30 °C, relative humidity: 40–60%) without any encapsulation, as shown in **Figure** **6**. It can be seen that the PCE of device with P3HT showed a clear degradation, which may be induced by the instability of P3HT under the ambient condition. But for HTM‐free device, the PCE showed no obvious degradation after 30 d. Compared with the initial PCE, the value of device without P3HT was slightly increased after five days storage. The enhanced PCE of devices was mainly due to the increase of *V*_oc_ (Figure S8, Supporting Information). The higher *V*_oc_ can be attributed to the less recombination according to the weaker PL intensity of film after five days storage than the initial one (Figure S9, Supporting Information). XRD pattern of the Cs_2_AgBiBr_6_ film showed no evidence of material decomposition under ambient condition after 30 d (Figure S10, Supporting Information), indicating good stability of this double perovskite film. For HTM‐free device, the PCE showed no obvious degradation after storing the device under ambient conditions for more than four months (Figure S11, Supporting Information). To the best of our knowledge, the stability of our double perovskite Cs_2_AgBiBr_6_‐based solar cells outperforms the typical hybrid APbX_3_ perovskite solar cell.^32^

**Figure 6:**
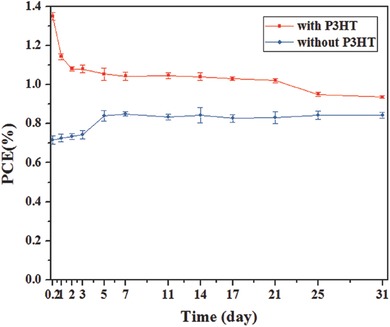
Stability of Cs_2_AgBiBr_6_ perovskite solar cells without encapsulation.

Though the PCE of our devices is relatively lower than Sn^2+^‐based lead‐free PSCs, the Sn^2+^ ion is unstable and easy to be oxidized to Sn^4+^, leading to a destruction of photovoltaic performance. The PCE of double perovskite Cs_2_AgBiBr_6_‐based solar cell is higher than the reported results of lead‐free (except Sn, which is fairly unstable) PSCs. Good intrinsic thermal stability together with moderate PCE provides the posibility to develop new lead‐free perovskite‐based solar cells. A summary of device performance of lead‐free (except Sn) PSCs is shown in **Table** **2**.

**Table 2:** Summary of device performance of other lead‐free (except Sn) lead‐free PSCs

Types	*E*_g_ [eV]	PCE [%]	*J*_sc_ [mA cm^−2^]	*V*_oc_ [V]	FF	Ref.
Cs_3_Bi_2_I_9_	2.20	1.09	2.15	0.85	0.60	31
MA_3_Bi_2_I_9_	2.10	0.12	0.52	0.68	0.33	31
MA_3_Bi_2_I_9_Cl*_x_*	2.40	<0.01	0.18	0.04	0.38	31
MA_3_Sb_2_I_9_	2.14	0.49	1.00	0.90	0.55	35
Rb_3_Sb_2_I_9_	2.24	0.66	2.11	0.55	0.56	36
(NH_4_)_3_Sb_2_I_9_	2.27	0.51	1.15	1.03	0.42	37
Cs_2_AgBiBr_6_	2.05	1.44	1.78	1.04	0.78	This work

Double perovskites are a new class of photovoltaic promising materials with good intrinsic stability. Through theory calculations, a PCE of 7.92%^33^ could be obtained for Cs_2_AgBiBr_6_. Future improvement can be carried on further device optimization and the bandgap engineering of the double perovskite. The energy alignment optimization of charge transporting materials should also be made to decrease the energy loss resulted from the interface between perovskite and charge transporting materials. For example, the highest occupied molecular orbital (HOMO) level of hole transporting material and the lowest unoccupied molecular orbital (LUMO) level of electron transporting material should be more close to those of perovskite. The value of exciton binding energy of 220 meV for Cs_2_AgBiBr_6_ is much larger than that of CH_3_NH_3_PbI_3_ (25 meV), which might also be one of the reasons for the lower power conversion efficiency of Cs_2_AgBiBr_6_ planar heterojunction device. Therefore, bulk heterojunction structure (like organic photovoltaic cell) might be used to improve the exciton separation. A suitable charge transporting material is necessary to mix with Cs_2_AgBiBr_6_ to form a uniform film, which can be heated to a high temperature (>250 °C). For bandgap engerneering, a direct bandgap of 1.57 eV^27^ could be obtained by adding 0.075% Tl (I) into Cs_2_AgBiBr_6_, and 1.86 eV^28^ for Cs_2_Ag(Bi_0.625_Sb_0.375_)Br_6_, which show great potential in solar cell applications. In addition, it was reported that the mono and trivalent metal ions in double perovskite have been substituted to form Cs_2_InBiCl_6_ or Cs_2_InSbCl_6_, which shows a direct energy gap of 0.91 and 1.02 eV.^22^ Therefore, it is promising to use double perovskite film as the absorption material, we can surely expect much more stable and efficient double perovskite solar cells in the future.

The double Cs_2_AgBiBr_6_ perovskite exhibits a good thermal stability, which is a significant requirement for photoelectric devices, and also shows a great potential for other devices, such as photodetector and transistor, could also be fabricated based on this high quality film.

In summary, we have successfully demonstrated a first high‐quality double perovskite Cs_2_AgBiBr_6_ film by a low‐pressure assisted solution method. More importantly, this film shows superior thermal and ambient stability. The planar heterojunction solar cell based on this Cs_2_AgBiBr_6_ film showed an optimal PCE of 1.44% at AM1.5 (100 mW cm^−2^) illumination, which is currently the top efficiency in lead‐free, highly stable perovskite solar cells. Our study opens the posibility that high quality double perovskite working layers can be successfully made. The results indicate that this material can be a promising candidate for a new type of lead‐free and highly stable perovskite solar cells.

## Experimental Section

*Materials*: SnO_2_ colloid precursor (tin (IV) oxide, 15% in H_2_O colloidal dispersion), anhydrous DMSO, BiBr_3_ (99%), 1,2‐dichlorobenzene were purchased from Alfa Aesar. CsBr (99.9%) and AgBr (99.99%) were purchased from Xi'an Polymer Light Technology Corp. (PLT) and Aladdin, respectively. P3HT was purchased from Rieke Metals. All these commercially available materials were used as received without any further purification.

*Cs_2_AgBiBr_6_ Preparation*: Cs_2_AgBiBr_6_ powder was prepared according to the procedure reported previously. Briefly, solid CsBr (1.696 g), AgBr (0.752 g), BiBr_3_ (1.792 g), and 40 mL HBr (48% in water) were reacted at 110 °C with stirring for 120 min. The solution was cooled to room temperature for overnight. Orange Cs_2_AgBiBr_6_ powder was obtained by rotary evaporation.

*Device Fabrication*: The indium tin oxide (ITO) glass was sequentially cleaned in deionized water, acetone, and ethanol under ultrasonic each for 30 min, and then treated with oxygen plasma for 10 min. The SnO_2_ electron transport layer was prepared by spin‐coating dispersed SnO_2_ colloid in deionized water (volume ratio of 1:6) at a speed of 3000 rpm, then annealed at 150 °C for 30 min. The double perovskite Cs_2_AgBiBr_6_ absorber layer was deposited by an LPA method under ambient condition. The Cs_2_AgBiBr_6_ powder was dissolved in DMSO to form a transparent light yellow precursor with a concentration of 0.5 mol L^−1^, then it was spin‐coated on the substrate at 2000 rpm. After that, the film was quickly moved to low pressure equipment followed by pumping to 20 Pa, then it was annealed at different temperatures for 10 min. When the substrate was cooled to room temperature, 60 µL P3HT (15 mg mL^−1^) 1,2‐dichlorobenzene solution was spin‐coated on the perovskite layer at 2000 rpm for 30 s. Finally, Au electrode was deposited by thermal evaporation at a rate of 0.3 nm s^−1^ using a shadow mask to pattern the electrode. The active area of solar cell is 0.1 cm^2^. Except for thermal evaporation, the whole process is carried out under ambient condition with a relative humidity (RH) of ≈50%.

*Characterization*: The XRD patterns were measured using X‐ray diffraction system (PANalytical Inc.) with monochromatic Cu Kα irradiation (λ = 1.5418 Å). UV–visible absorption spectrum was measured by using a UV–vis–NIR spectrophotometer (UV3600Plus). The maximum valence band of Cs_2_AgBiBr_6_ film was identified by ultraviolet photoelectron spectroscopy (Axis Ultra). SEM images were recorded by using a high‐resolution scanning electron microscopy (Hitachi S‐4800). PL (excited at 450 nm) was measured with NaonLog infrared fluorescence spectrometer (Nanolog FL3‐2Ihr). TRPL measurement was measured using UltraFast lifetime Spectrometer (Delta flex). Referring to previous method,^34^ photovoltaic performances were measured by using a Keithley 2611 source meter at AM 1.5G illumination (100 mW cm^−2^) under a Newport Thermal Oriel 69911 300 W solar simulator. The system was calibrated against a certified reference silicon solar cell. Effective area of each cell was 0.1 cm^2^ defined by masks for all the photovoltaic devices discussed in this work. The rate of *J*–*V* scan is 0.13 V s^−1^ with a sweep delay of 100 ms.

## Conflict of Interest

The authors declare no conflict of interest.

## Supporting information

Supplementary
