# Supplementary material for: The Dawn of Lead‐Free Perovskite Solar Cell: Highly Stable Double Perovskite Cs2AgBiBr6 Film
Source: Adv Sci (Weinh). 2017 Dec 18;5(3):1700759. doi: 10.1002/advs.201700759 (PMC5867041; doi:10.1002/advs.201700759)
Supplement: Supplementary file 1 — Supplementary [file ADVS-5-1700759-s001.pdf]

Copyright WILEY-VCH Verlag GmbH & Co. KGaA, 69469 Weinheim, Germany, 2017.

ADVANCED SCIENCE
Open Access

# Supporting Information

for *Adv. Sci.,* DOI: 10.1002/advs.201700759

# The Dawn of Lead-Free Perovskite Solar Cell: Highly Stable Double Perovskite $Cs_2AgBiBr_6$ Film

*Cuncun Wu, Qiaohui Zhang, Yang Liu, Wei Luo, Xuan Guo, Ziru Huang, Hungkit Ting, Weihai Sun, Xinrui Zhong, Shiyuan Wei, Shufeng Wang,* Zhijian Chen,* and Lixin Xiao**

Supporting Information

# The dawn of lead-free perovskite solar cell: Highly stable double perovskite $Cs_2AgBiBr_6$ film

*Cuncun Wu, Qiaohui Zhang, Yang Liu, Wei Luo, Xuan Guo, Ziru Huang, Hungkit Ting, Weihai Sun, Xinrui Zhong, Shiyuan Wei, Shufeng Wang*, Zhijian Chen*, Lixin Xiao**

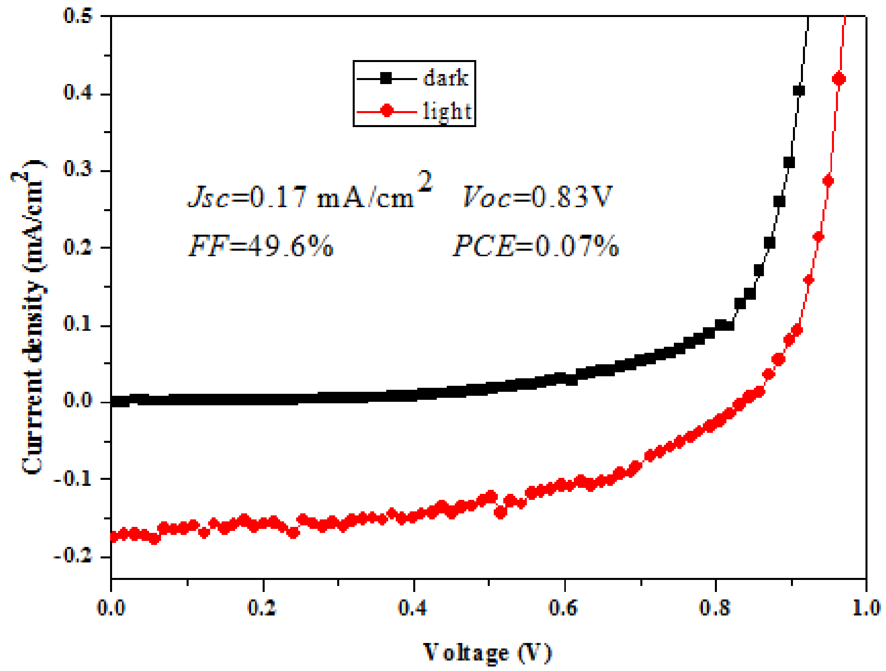


**Figure S1** Current-voltage (J-V) characteristics of SA $Cs_2AgBiBr_6$ based solar cells.

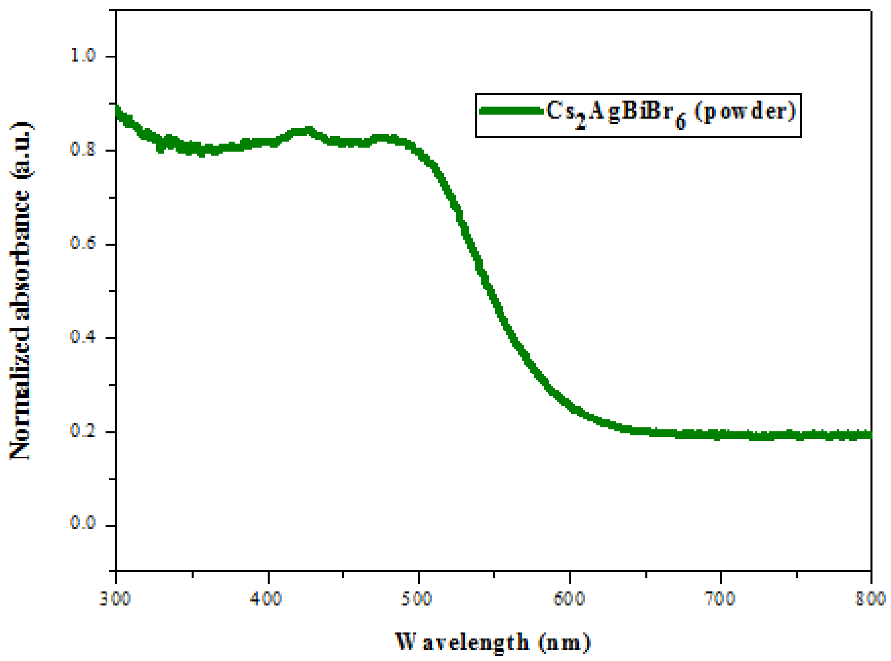


**Figure S2** Absorption spectrum of $Cs_2AgBiBr_6$ powder.

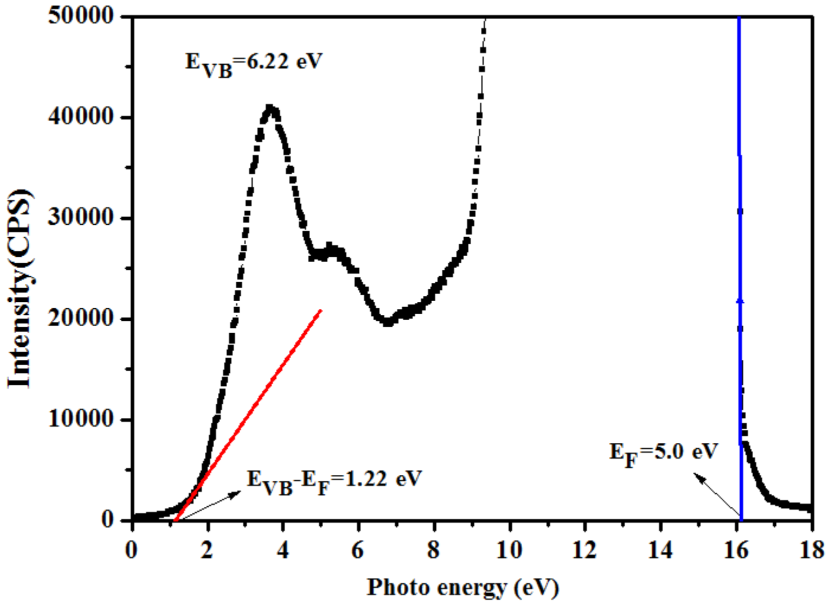


**Figure S3** ultraviolet photoelectron spectrum of $Cs_2AgBiBr_6$ film .

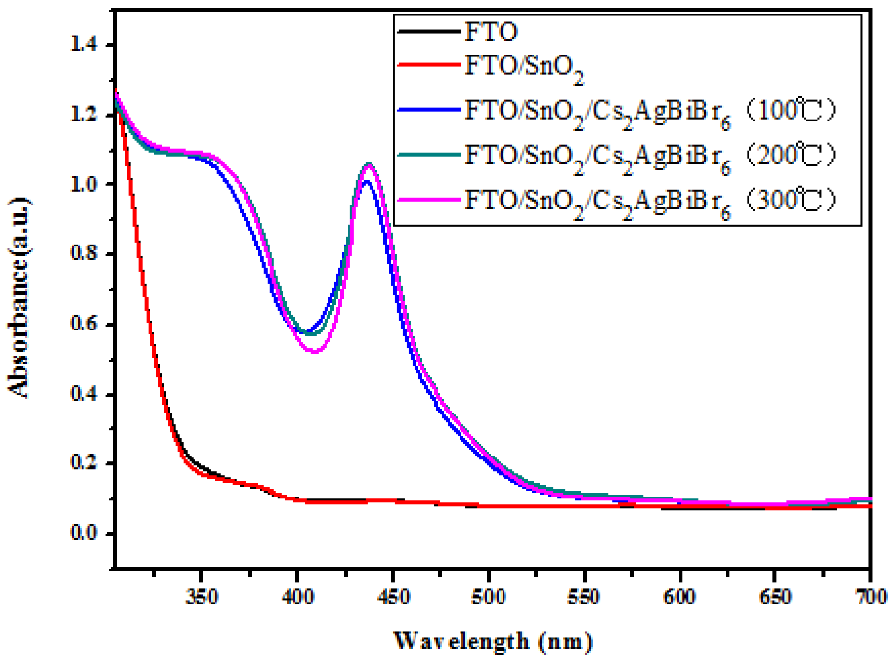


**Figure S4** Absorption spectrum of $Cs_2AgBiBr_6$ film from different annealing temperatures.

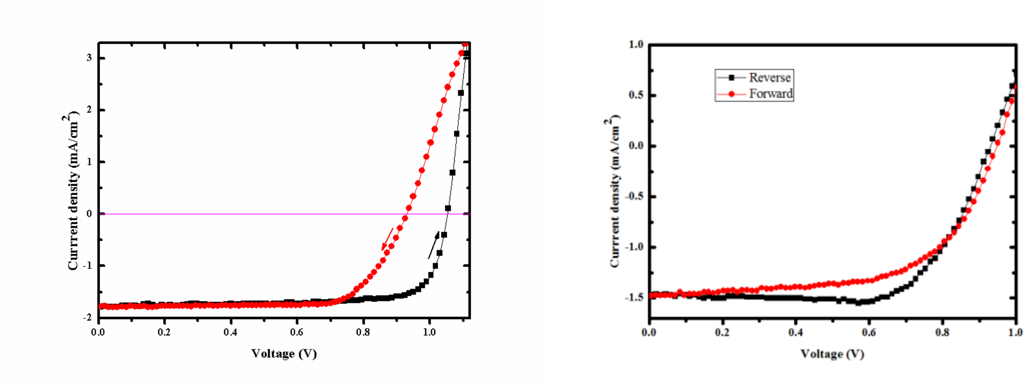


**Figure S5** J-V characteristics of device with (left) or without (right) P3HT at forward (red) and reverse (black) scan direction.

**Table S1.** Photovoltaic performances of the cell with or without P3HT at forward and reverse scan direction.

| Sample | $J_{SC}$(mA/cm$^2$) | $V_{OC}$ (V) | FF | PCE(%) |
|---|---|---|---|---|
| $Cs_2AgBiBr_6$ /P3HT(Forward) | 1.78 | 1.04 | 0.78 | 1.44 |
| $Cs_2AgBiBr_6$/P3HT (Reverse) | 1.78 | 0.93 | 0.73 | 1.21 |
| $Cs_2AgBiBr_6$ (Forward) | 1.47 | 0.94 | 0.62 | 0.85 |
| $Cs_2AgBiBr_6$ (Reverse) | 1.47 | 0.93 | 0.71 | 0.97 |

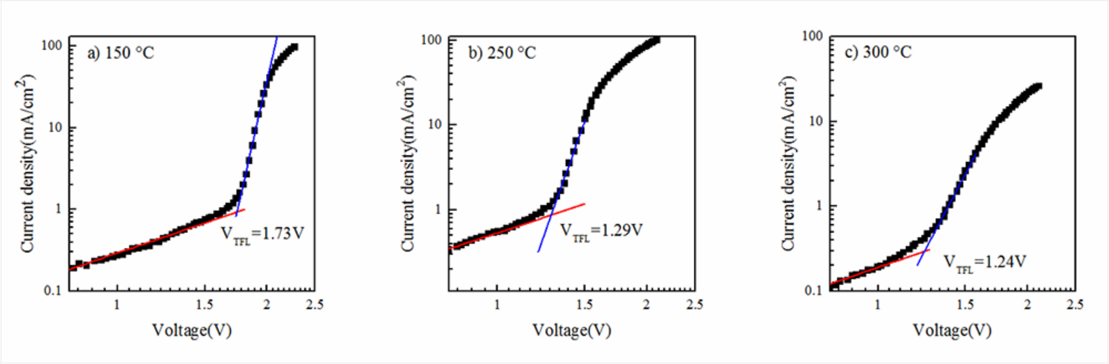


**Figure S6** J-V curves of the ITO/$Cs_2AgBiBr_6$/Au device under dark condition.

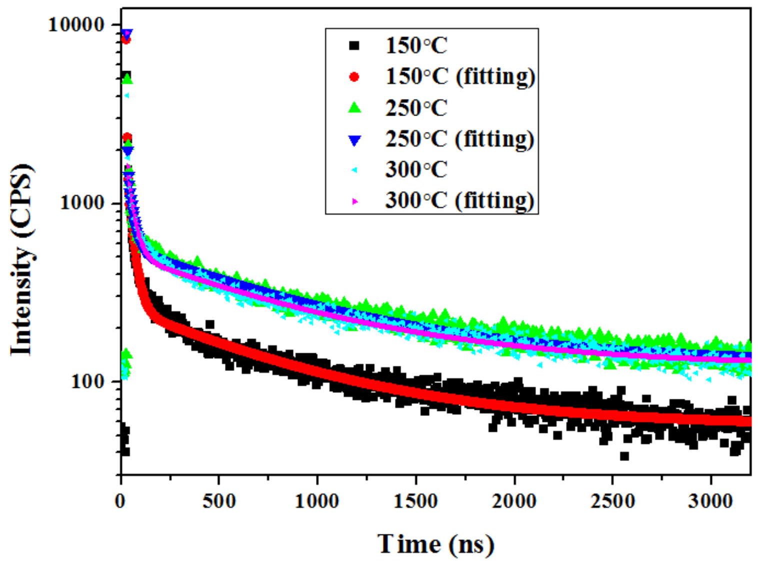


**Figure S7** time resolved photoluminescence of $Cs_2AgBiBr_6$ film deposited on quartz glass with different annealing temperatures.

**Table S2.** time resolved photoluminescence results of $Cs_2AgBiBr_6$ film with different annealing temperatures.

| Sample | $\tau_1$(ns) | $\tau_1$ ratio(%) | $\tau_2$ | $\tau_2$ ratio(%) | $\tau_3$ | $\tau_3$ ratio(%) |
|---|---|---|---|---|---|---|
| 150℃ | 2.17 | 7.12 | 29.9 | 18.63 | 771.8 | 74.26 |
| 250℃ | 1.46 | 2.65 | 25.3 | 7.5 | 843.6 | 89.85 |
| 300℃ | 0.97 | 1.91 | 32.6 | 10.03 | 838.9 | 88.06 |

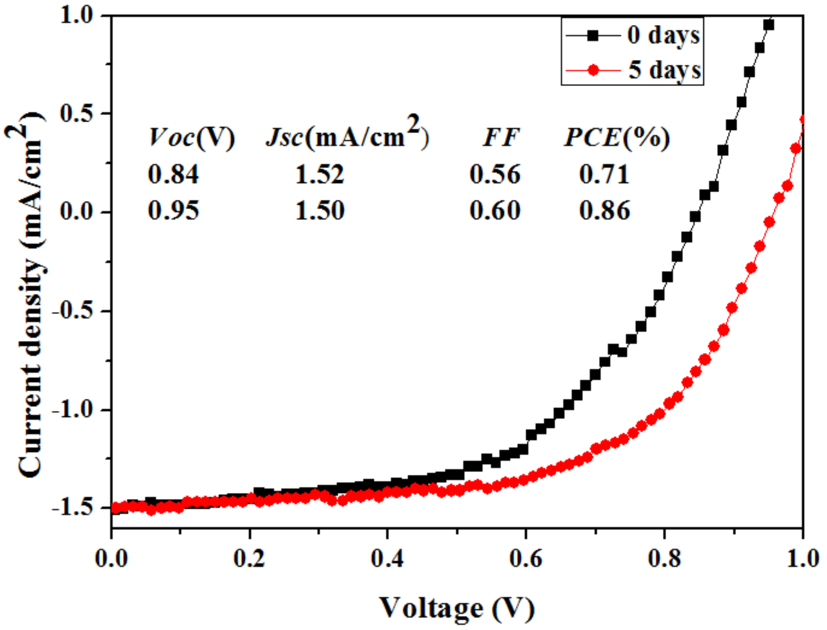


**Figure S8** J-V characteristics of the intial cell and with five days storage in air for the device without P3HT

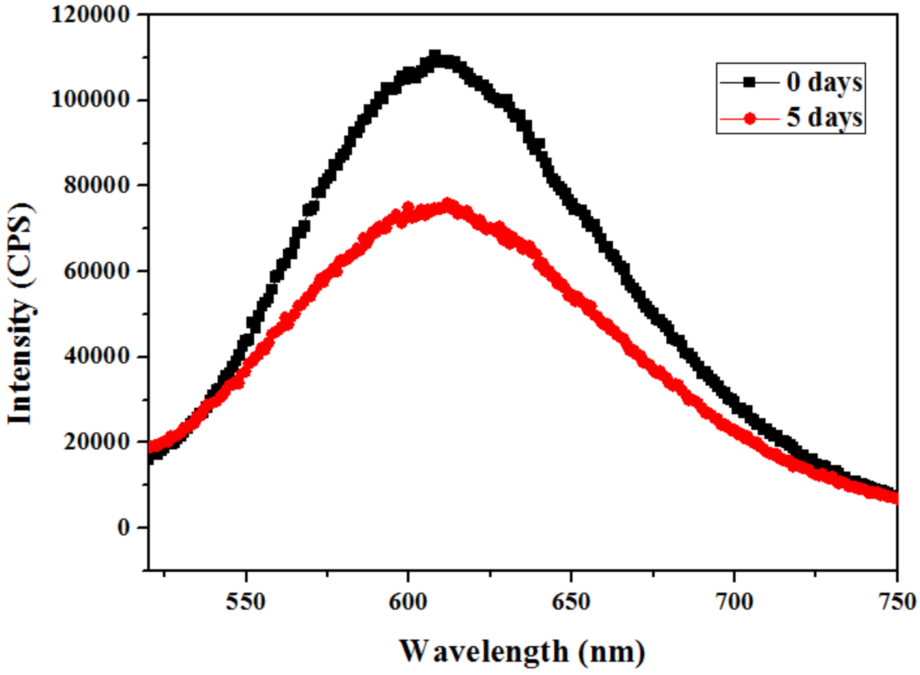


**Figure S9** Steady-state photoluminescence of $Cs_2AgBiBr_6$ film and the film with five days storage in air.

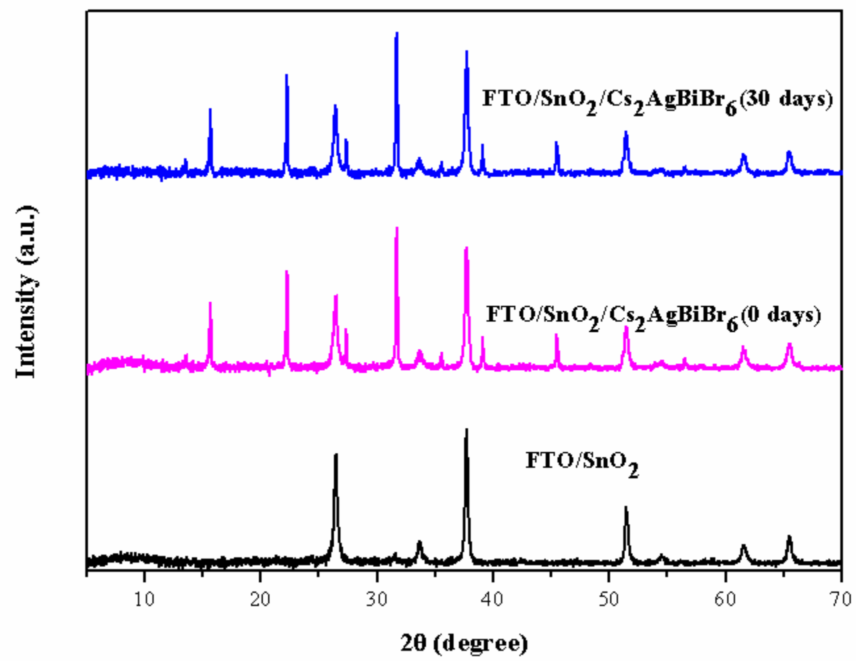


**Figure S10** XRD pattern of $Cs_2AgBiBr_6$ film under ambient condition for 30 days.

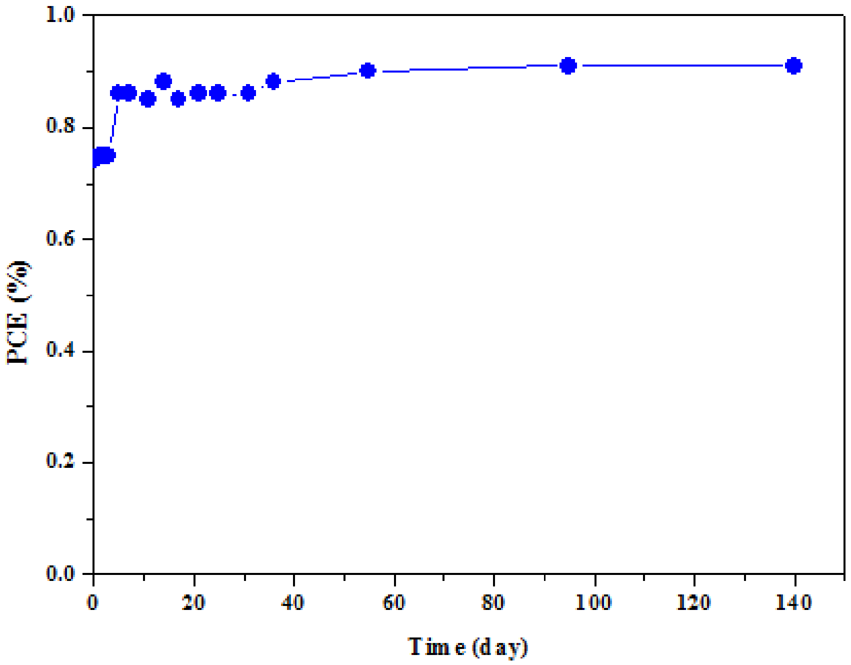


**Figure S11** Long time stability of HTM free device under ambient condition.
